# Radiation Necrosis Following Stereotactic Radiosurgery or Fractionated Stereotactic Radiotherapy with High Biologically Effective Doses for Large Brain Metastases

**DOI:** 10.3390/biology12050655

**Published:** 2023-04-26

**Authors:** Leonie Johannwerner, Elisa M. Werner, Oliver Blanck, Stefan Janssen, Florian Cremers, Nathan Y. Yu, Dirk Rades

**Affiliations:** 1 Department of Radiation Oncology, University of Lubeck, 23562 Lubeck, Germany; leonie.johannwerner@web.de (L.J.); elisamarie.werner@uksh.de (E.M.W.); stefan.janssen@uksh.de (S.J.); florian.cremers@uksh.de (F.C.); 2 Department of Radiation Oncology, University Medical Center Schleswig-Holstein, Campus Kiel, 24105 Kiel, Germany; oliver.blanck@uksh.de; 3 Saphir Radiosurgery Center Northern Germany, 24105 Kiel, Germany; 4 Medical Practice for Radiotherapy and Radiation Oncology, 30161 Hannover, Germany; 5 Department of Radiation Oncology, Mayo Clinic, Phoenix, AZ 85054, USA; yu.nathan@mayo.edu

**Keywords:** brain metastasis, radiation necrosis, stereotactic radiosurgery, fractionated stereotactic radiotherapy, biologically effective dose

## Abstract

**Simple Summary:**

Based on Radiation Therapy Oncology Group (RTOG) 90-05, a maximum biologically effective dose (BED) of 45 Gy_12_ is recommended for the stereotactic radiosurgery (SRS) of brain metastases measuring 21–30 mm. Given that patients on RTOG 90-05 received prior brain irradiation, the tolerable BED for de novo lesions may be >45 Gy_12_. We investigated radiation necrosis (RN) after single-fraction SRS or fractionated stereotactic radiotherapy (FSRT) with BED > 49 Gy_12_ for 1–4 radiotherapy-naïve brain metastases. In the entire cohort (169 patients with 218 lesions) and in patients with all brain metastases ≤ 20 mm (137 patients with 185 lesions), 1-year and 2-year RN rates were not significantly different after SRS or FSRT. In patients with metastases > 20 mm (32 patients with 33 lesions), the RN rates were significantly higher after SRS in both per-patient and per-lesion analyses. Moreover, in the SRS group, lesion size > 20 mm was significantly associated with RN. FSRT with BED > 49 Gy_12_ was associated with low RN risk for metastases > 20 mm and appeared to be safer than SRS for such lesions.

**Abstract:**

In Radiation Therapy Oncology Group 90-05, the maximum tolerated dose of single-fraction radiosurgery (SRS) for brain metastases of 21–30 mm was 18 Gy (biologically effective dose (BED) 45 Gy_12_). Since the patients in this study received prior brain irradiation, tolerable BED may be >45 Gy_12_ for de novo lesions. We investigated SRS and fractionated stereotactic radiotherapy (FSRT) with a higher BED for radiotherapy-naive lesions. Patients receiving SRS (19–20 Gy) and patients treated with FSRT (30–48 Gy in 3–12 fractions) with BED > 49 Gy_12_ for up to 4 brain metastases were compared for grade ≥ 2 radiation necrosis (RN). In the entire cohort (169 patients with 218 lesions), 1-year and 2-year RN rates were 8% after SRS vs. 2% and 13% after FSRT (*p* = 0.73) in per-patient analyses, and 7% after SRS vs. 7% and 10% after FSRT (*p* = 0.59) in per-lesion analyses. For lesions ≤ 20 mm (137 patients with 185 lesions), the RN rates were 4% (SRS) vs. 0% and 15%, respectively, (FSRT) (*p* = 0.60) in per-patient analyses, and 3% (SRS) vs. 0% and 11%, respectively, (FSRT) (*p* = 0.80) in per-lesion analyses. For lesions > 20 mm (32 patients with 33 lesions), the RN rates were 50% (SRS) vs. 9% (FSRT) (*p* = 0.012) in both per-patient and per-lesion analyses. In the SRS group, a lesion size > 20 mm was significantly associated with RN; in the FSRT group, lesion size had no impact on RN. Given the limitations of this study, FSRT with BED > 49 Gy_12_ was associated with low RN risk and may be safer than SRS for brain metastases > 20 mm.

## 1. Introduction

Many patients with brain metastases receive stereotactic radiotherapy, either alone or in combination with whole-brain radiotherapy [1,2,3,4]. Stereotactic radiotherapy can be administered as single-fraction stereotactic radiosurgery (SRS) or as fractionated stereotactic radiotherapy, often including three or five daily fractions (FSRT). Stereotactic radiotherapy has advantages over whole-brain radiotherapy, including the preservation of neurocognitive function [5,6]. However, the use of stereotactic radiotherapy for brain metastases is limited by the size (maximum diameter) and total volume of the lesions [1,2,3,4]. It is recommended that the biologically effective dose (BED) of SRS should be adapted to the size of the metastatic lesions. In Radiation Therapy Oncology Group (RTOG) 90-05, the maximum tolerated doses of SRS for larger brain metastases were 18 Gy for lesions of 21–30 mm and 15 Gy for lesions of 31–40 mm, respectively [7]. The BEDs of these doses were 45 Gy_12_ and 33.8 Gy_12_, respectively. 

The BED allows one to compare different dose-fractionation regimens of radiotherapy with respect to the biological effect regarding tumor control and is calculated using the formula BED = TD × (1 + DFx/α/β). TD means total dose, DFx means dose per fraction, and α/β stands for *alpha/beta* ratio, which is 12 Gy for the irradiation of brain metastases [8,9,10]. To make it clear that the BED has been calculated with an *alpha/beta* ratio of 12 Gy, the BED is given as Gy_12_ instead of Gy. Many, and radiation oncologists follow the recommendations of the RTOG 90-05 study [7]. However, the RTOG study enrolled patients who received prior radiation to the brain with median 60 Gy (primary brain tumors) or 30 Gy (brain metastases). Thus, the maximal tolerated BED for de novo brain metastases may be greater than 45 Gy_12_.

Radiation necrosis (RN) represents an important dose-limiting complication after SRS or FSRT, which generally occurs several months or even years following radiotherapy [7]. Two main theories exist regarding the pathophysiology of RN, although it is likely multifactorial [11]. One theory suggests that stereotactic radiotherapy leads to the damage of the blood–brain barrier with subsequently increased capillary leakage and vascular permeability [11]. The activation of acid sphingomyelinase and the up-regulation of ceramide lead to endothelial apoptosis, an increase in oxygen free radicals, and the production of vascular endothelial growth factor and intercellular adhesion molecules. Finally, these processes result in stenosis and fibrinoid necrosis of small blood vessels, and consequently ischemia and cell death. The second theory involves radiotherapy, leading to the demyelination of glial cells [11]. Damage to endothelial cells results in the release of vascular endothelial growth factor and hypoxia-inducible factor 1 alpha. Capillaries induced by neo-angiogenesis are permeable, which leads to edema and contrast extravasation.

The prevalence of RN ranges between 3% and 34% [7,9,12,13,14,15,16,17,18,19,20,21,22]. Symptomatic RN was reported to occur in up to 16% of patients after stereotactic radiotherapy [12,14,15,17,18,19,20,21]. The risk of RN increases with the size of the irradiated lesions and the BED of the radiation treatment [7,9,12,13,14,15]. We investigate and compare the prevalence of grade ≥ 2 (symptomatic) RN after SRS or FSRT with a higher BED (>49 Gy_12_) for radiotherapy-naive brain metastases with a particular focus on larger lesions > 20 mm.

## 2. Patients and Methods

The data of 169 patients who received SRS or FSRT alone (without WBRT) with BED > 49 Gy_12_ for up to 4 brain metastases (218 irradiated lesions in total) between 2011 and 2022 were retrospectively analyzed. SRS (CyberKnife^®^) was performed by the Saphir Radiosurgery Center Northern Germany with 20 Gy (BED = 53.3 Gy_12_) in 93 patients and 19 Gy (BED = 49.1 Gy_12_), prescribed to the 60–80% isodose line. Gross tumor volume (GTV) represented the planning target volume (PTV). FSRT was performed by the Department of Radiation Oncology of the University of Lübeck or by the Medical Practice for Radiotherapy and Radiation Oncology Hannover with a linear accelerator, including the ExacTrac^®^ positioning system. Doses of FSRT were prescribed to the 80% isodose line (PTV = GTV + 2 mm). Dose-fractionation included 3 × 10 Gy in 13 patients (BED = 55.0 Gy_12_), 3 × 11 Gy in 22 patients (BED = 63.3 Gy_12_), 5 × 7 Gy in 1 patient (BED = 55.4 Gy_12_), 5 × 8 Gy in 3 patients (BED = 66.7 Gy_12_), 6 × 6 Gy in 1 patient (BED = 54.0 Gy_12_), 7 × 5 Gy in 3 patients (BED = 49.6 Gy_12_), 7 × 6 Gy in 1 patient (BED = 63.0 Gy_12_), 7 × 6.25 Gy in 1 patient (BED = 66.5 Gy_12_), 8 × 5 Gy in 1 patient (BED = 56.7 Gy_12_), 9 × 5 Gy in 2 patients (BED = 63.8 Gy_12_), 10 × 4 Gy in 20 patients (BED = 53.3 Gy_12_), and 12 × 4 Gy in 1 patient (BED = 64.0 Gy_12_). The reasons for the use of different dose-fractionation regimens included the treating center, the period of time (e.g., three-fraction regimens relatively often used until 2015), the type of primary tumor (a higher BED for metastases from less radiosensitive tumors such as melanoma and renal cell carcinoma), size of metastatic lesions (tendency to use a higher BED for smaller lesions), and metastatic sites (a lower BED for lesions close to the brain stem, optic chiasm, and other organs at risk). Regarding the number of brain metastases, 129 patients had 1 lesion, 26 patients had 2 lesions, 10 patients had 3 lesions, and 4 patients had 4 lesions, respectively. Regarding the primary tumor types, 71 patients had lung cancer, 58 patients had melanoma, and 40 patients had other primary tumor types. Other types included breast cancer (20 patients), colorectal cancer (6 patients), kidney cancer (5 patients), cancer of the upper gastrointestinal tract (5 patients), bladder cancer (2 patients), thyroid cancer (1 patient), and cancer of unknown primary (1 patient).

The distributions of the numbers of brain metastases, the primary tumor types, and other characteristics in the SRS group and the FSRT group are shown in Table 1. A total of 32 patients had at least 1 metastasis with a maximum diameter of >20 mm, which was 21–30 mm in 27 patients, 31–40 mm in 4 patients, and >40 mm in 1 patient. A total of 11 patients in this group received SRS with 20 Gy (9 patients) or 19 Gy (2 patients). A total of 21 patients received FSRT with 3 × 10 Gy (3 patients), 3 × 11 Gy (4 patients), 7 × 6 Gy (1 patient), 9 × 5 Gy (1 patient), 10 × 4 Gy (11 patients), or 12 × 4 Gy (1 patient).

SRS and FSRT were compared with respect to symptomatic (grade ≥ 2) RN at 1 year and 2 years after radiotherapy in the entire cohort (169 patients), and additionally in the 32 patients with at least 1 metastasis > 20 mm (characteristics given in Table 2) and the 137 patients where all metastases were ≤20 mm (characteristics given in Table 3). In the entire patient cohort, additional factors were evaluated for associations with RN, including the biologically effective dose (49–57 Gy_12_ vs. 63–67 Gy_12_), year of treatment (2011–2016 vs. 2017–2022), systemic treatment within 6 months prior to stereotactic radiotherapy for brain metastases (no vs. yes), age (≤median vs. >median age; median age was 66 years in the entire cohort and in patients with all metastases ≤ 20 mm, and 65 years in patients with at least 1 metastasis >20 mm), gender (female vs. male), Karnofsky performance score (KPS ≤80 vs. ≥90), primary tumor type (lung cancer vs. melanoma vs. other types), number of brain metastases (1 vs. 2–4), main metastatic site(s) (supratentorial–peripheral vs. supratentorial–central vs. infratentorial), maximum size of brain metastases (≤20 vs. >20 mm), and metastases outside the brain (no vs. yes) (Table 4). The potential impact of the year of treatment was evaluated, because an increasing use of modern targeted therapies likely had an impact on the treatment outcomes [15,16,17,18,19,20,21,23,24,25]. Since 2017, the use of checkpoint inhibitors has particularly increased [16,18,19,20,24,25]. In addition, separate analyses were performed in the SRS group and the FSRT group, comparing lesions ≤ 20 vs. >20 mm in terms of RN rates (Table 5). Moreover, the RN rates were calculated in a per-lesion manner in the entire cohort (218 lesions) and specific subgroups regarding radiotherapy approach and lesion size (Table 6).

The Kaplan–Meier method and the log-rank test were used for univariate analyses of RN, and the Cox proportional hazards model was applied for multivariate analyses including factors found to be significantly associated with RN (*p* < 0.05) via univariate analyses (BlueSky Statistics 10 GA, BlueSky Statistics LLC, Chicago, IL, USA). Distributions of the patient characteristics were compared between the SRS and FSRT groups in the entire cohort (Table 1), in patients with lesions > 20 mm (Table 2), and in patients with all lesions ≤ 20 mm (Table 3) using the chi-square test or Fisher’s exact test (less than 5 patients). *P*-values < 0.05 indicated significance.

## 3. Results

The median follow-up times were 12 months (range: 1–93 months) in the entire cohort and 21 months (range: 4–93 months) in those patients who were alive at the last follow-up contact. In the entire cohort, significantly more patients receiving SRS compared to patients treated with FSRT had a KPS ≥ 90 (*p* < 0.001), only 1 brain metastasis (*p* < 0.001), or brain metastases ≤ 20 mm in maximum diameter (*p* = 0.002); received systemic therapy prior to radiotherapy of brain metastasis (*p* = 0.004); and were treated with a BED of 49–57 Gy_12_ (*p* < 0.001) (Table 1). These differences were also found in all patients with brain metastases ≤ 20 mm (Table 3). In the group of patients with at least 1 brain metastasis >20 mm, KPS was the only characteristic with a significantly different distribution between the SRS and FSRT groups (Table 2).

**Figure 1:**
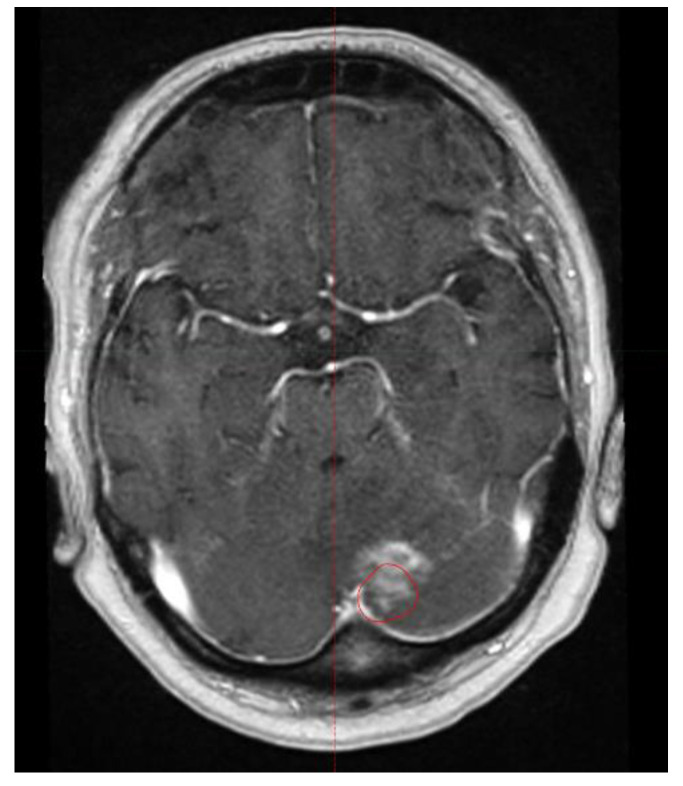
Example of radiation necrosis that occurred 20 months following fractionated stereotactic radiotherapy (FSRT). The red line represents the former planning target volume.

In the entire patient cohort, the occurrence of RN was significantly associated with the maximum size of brain metastases (*p* = 0.0495), whereas the type of radiotherapy (SRS vs. FSRT) did not show such an association (*p* = 0.72) (Table 4). In the Cox proportional hazards model, the size of brain metastases showed a trend (hazard ratio: 1.92; 95% confidence interval: 0.97–3.42; *p* = 0.064). An example of RN is given in Figure 1.

**Table 4:** Rates of symptomatic radiation necrosis in the entire patient cohort (*n* = 169).

Characteristic	Radiation Necrosis Rate		*p*-Value
	At 1 Year (in %)	At 2 Years (in %)	
Type of radiotherapy			0.73
Stereotactic radiosurgery	8	8	
Fractionated stereotactic radiotherapy	2	13	
Biologically effective dose			0.14
49–57 Gy_12_	5	5	
63–67 Gy_12_	5	26	
Year of treatment			0.082
2011–2016	7	14	
2017–2022	4	7	
Pre-RT systemic treatment			0.37
No	5	15	
Yes	6	6	
Age at RT			0.35
≤66 years	6	14	
≥67 years	5	5	
Gender			0.75
Female	4	10	
Male	7	11	
Karnofsky performance score			0.63
≤80	0	13	
≥90	8	10	
Type of primary tumor			0.53
Lung cancer	8	18	
Melanoma	3	6	
Other types	4	4	
Number of brain metastases			0.99
1	5	10	
2–4	6	13	
Main metastatic site(s)			0.65
Supratentorial–peripheral	6	9	
Supratentorial–central	20	20	
Infratentorial	0	8	
Maximum size of brain metastases			**0.0495**
≤20 mm	2	9	
>20 mm	20	20	
Metastases outside the brain			0.92
No	5	10	
Yes	5	10	

RT: radiotherapy.

In the subgroup analysis of patients with at least 1 metastasis > 20 mm, the occurrence of RN was significantly associated with SRS (vs. FSRT) in the univariate analysis (*p* = 0.012) and the subsequent multivariate analysis (hazard ratio: 0.10; 95% confidence interval: 0.01–0.94; *p* = 0.044). Both 1-year and 2-year RN rates were 50% after SRS vs. 9% after FSRT. In all patients with brain metastases ≤ 20 mm, the type of radiotherapy had no significant impact on RN. The 1-year and 2-year RN rates were both 4% after SRS vs. 0% and 15%, respectively, after FSRT (*p* = 0.60). These results are summarized in Table 5.

**Table 5:** Rates of symptomatic radiation necrosis in the investigated subgroups (per-patient analyses).

Subgroup	Radiation Necrosis Rate		*p*-Value
	At 1 Year (in %)	At 2 Years (in %)	
**Patients with ≥1 metastasis >20 mm**			**0.012**
Stereotactic radiosurgery	50	50	
Fractionated stereotactic radiotherapy	9	9	
**Patients with all metastases ≤ 20 mm**			0.60
Stereotactic radiosurgery	4	4	
Fractionated stereotactic radiotherapy	0	15	
**Stereotactic radiosurgery**			**<0.001**
Size of metastases ≤ 20 mm	4	4	
Size of ≥1 metastasis >20 mm	50	50	
**Fractionated stereotactic radiotherapy**			0.93
Size of metastases ≤ 20 mm	0	15	
Size of ≥1 metastasis >20 mm	9	9	

In the separate analysis of RN in the SRS group, the RN rates were significantly higher with lesions > 20 mm (*n* = 89) vs. ≤20 mm (*n* = 48) as found via the univariate analysis (50% vs. 4% at both 1 year and 2 years, *p* < 0.001) and multivariate analysis (hazard ratio: 3.82; 95% confidence interval: 1.70–8.58, *p* = 0.001). In the FSRT group, the maximum size of the brain metastases had no significant impact (*p* = 0.93) on the RN rates at 1 year (9% vs. 0%) and 2 years (9% vs. 15%). These results are also summarized in Table 5.

In the additional analyses performed in a per-lesion manner in the entire cohort (218 lesions), the RN rates were significantly higher after radiotherapy of lesions > 20 mm in the univariate (*p* = 0.020, Table 6) and the multivariate analyses (hazard ratio: 4.03; 95% confidence interval: 1.13–14.32; *p* = 0.031). The RN rates after SRS and FSRT were not significantly different (Table 6). In the subgroup analyses, higher RN rates were significantly associated with SRS (vs. FSRT) in patients with lesions > 20 mm in the univariate (*p* = 0.012, Table 6) and the multivariate analyses (hazard ratio: 0.10; 95% confidence interval: 0.01–0.94; *p* = 0.044). Moreover, RN was positively associated with a lesion size > 20 mm in patients receiving SRS in the univariate (*p* < 0.001, Table 6) and the multivariate analyses (hazard ratio: 16.56; 95% confidence interval: 3.30–83.20; *p* < 0.001).

**Table 6:** Rates of symptomatic radiation necrosis in the entire cohort and in the investigated subgroups (per-lesion analyses).

Group	Radiation Necrosis Rate		*p*-Value
	At 1 Year (in %)	At 2 Years (in %)	
**Entire cohort**			0.59
Stereotactic radiosurgery (118 lesions)	7	7	
Fractionated stereotactic RT (100 lesions)	2	10	
**Entire cohort**			**0.020**
Size of metastases ≤ 20 mm (185 lesions)	2	7	
Size of ≥1 metastasis >20 mm (33 lesions)	20	20	
**At least 1 lesion >20 mm**			**0.012**
Stereotactic radiosurgery (11 lesions)	50	50	
Fractionated stereotactic RT (22 lesions)	9	9	
**All lesions ≤ 20 mm**			0.80
Stereotactic radiosurgery (107 lesions)	3	3	
Fractionated stereotactic RT (78 lesions)	0	11	
**Stereotactic radiosurgery**			**<0.001**
Size of metastases ≤ 20 mm (107 lesions)	3	3	
Size of ≥1 metastasis >20 mm (11 lesions)	50	50	
**Fractionated stereotactic RT**			0.86
Size of metastases ≤ 20 mm (78 lesions)	0	11	
Size of ≥1 metastasis >20 mm (22 lesions)	9	9	

RT: radiotherapy.

## 4. Discussion

Many patients with brain metastases receive stereotactic radiotherapy (SRS or FSRT) [1,2,3,4]. It is generally recommended that the BED of stereotactic radiotherapy be adapted to the maximum diameter of the metastatic lesions [7]. In RTOG 90-05, the maximum tolerated doses of SRS for lesions of ≤20 mm, 21–20 mm, and 31–40 mm were 24 Gy, 18 Gy, and 15 Gy, respectively. These doses represented BEDs of 72 Gy_12_, 45 Gy_12_, and 33.8 Gy_12_, respectively. We felt that a BED > 45 Gy_12_ may also be safely delivered for brain metastases > 20 mm, particularly if stereotactic radiotherapy is delivered as FSRT. RN is a significant dose-limiting toxicity of SRS or FSRT [7]. Therefore, we investigated grade ≥ 2 RN after SRS or FSRT with a higher BED (>45 Gy_12_) for brain metastases > 20 mm. Our hypothesis was that stereotactic radiotherapy with a higher BED might be feasible for larger lesions.

A meta-analysis of 24 trials from 2019 and a systematic review of 15 studies from 2021 have already evaluated the role of SRS and FSRT for larger brain metastases of ≥20 mm and >20 mm, respectively [26,27]. The authors of the meta-analysis concluded that FSRT may lead to a relative resection of RN [26]. In contrast to our present study, metastases of 20 mm with the largest diameter were included in that meta-analysis. Moreover, only 17 of the 24 trials included unresected lesions. In 8 of these 17 trials, patients received WBRT prior to SRS, and 4 other trials did not report whether previous WBRT was given [26]. Four of the remaining five trials reported data regarding RN, but only two of these trials compared SRS and FSRT like we did in the present study [14,28]. The systematic review of fifteen studies [27] included these two trials plus one other study which compared SRS and FSRT alone for unresected larger brain metastases [14,28,29]. In comparison to our study, the three previous studies used SRS and FSRT with a lower BED. SRS doses ranged between 14 Gy and 18 Gy (BED = 30.3–45.0 Gy_12_), and the FSRT regimens were 3 × 7.7 Gy, 3 × 8 Gy, and 3 × 9 Gy, respectively (BED = 37.9–47.3 Gy_12_) [14,28,29]. Thus, the present study is different from previous ones and, therefore, can be considered to be unique.

Additionally, indeed, FSRT with a BED > 45 Gy_12_ appeared to be safe for lesions > 20 mm in terms of symptomatic RN. The rate of 9% after 2 years in both per-patient and per-lesion analyses was within the range of 4–15%, found in previous studies to not be limited to brain metastases > 20 mm [12,15,17,18,19,20]. In a prospective observational study of 206 consecutive patients treated for 1–3 brain metastases with linear-accelerator-based SRS, 24% of the patients experienced RN, which was symptomatic in 10% of the patients [12]. SRS doses were 20 Gy for lesions ≤ 20 mm, and 18 Gy or 15–16 Gy for larger metastases or lesions in the brainstem. In a recent retrospective study, 412 patients received SRS (doses not stated) for cerebral lesions (median size: 20 mm) [15]. After a median of 10 months, 17% of the patients developed RN of any grade with 65% of these patients being symptomatic. However, the cohort investigated in this study was heterogeneous. Only 82% of the patients were treated for malignant disease, and some patients received prior neurosurgical resection or WBRT. Therefore, the comparison of this study to the results of our study appears to be limited. When compared to a study of 37 patients receiving FSRT with a median total dose of 35 Gy (range 30–41 Gy) given in 3 to 5 fractions for a total of 38 large brain metastases (>30 mm), the rate of grade ≥ 2 RN in our study was lower (9% vs. 16%) [21].

In 4 other retrospective studies that investigated SRS plus the administration of systemic therapies (immune checkpoint inhibitors in 3 studies an different therapies in 1 study), symptomatic RN occurred in 4% to 15% of the patients [17,18,19,20]. In these studies, systemic therapies may have contributed to the development of RN, which was shown in several studies, particularly for immunotherapy [16,17,23]. However, other studies did not find an increased rate of RN when systemic therapies were added to stereotactic radiotherapy [24,25]. In our study, systemic treatment within 6 months prior to stereotactic radiotherapy also had no significant impact on RN. Thus, the impact of additional systemic therapies on RN requires further clarification. In addition to our present study, 1 other study focused on brain metastases > 20 mm [14]. In this study, the cumulative 1-year RN rate was 9% after FSRT with 3 × 9 Gy (BED = 47.3 Gy_12_). The authors do not mention the 1-year rate of symptomatic RN but state that 4 of the 138 patients receiving FSRT developed symptomatic RN requiring surgery or medical treatment. This would correspond to a rate of 3%, which is lower than in the present study.

In our study, SRS was associated with significantly higher rates of grade ≥ 2 RN at 1 year and 2 years when compared to FSRT. Moreover, the RN rates after SRS were much higher than those observed in the previous study by Minniti et al. after SRS with 15–18 Gy for brain metastases > 20 mm (50% vs. 9%) [14]. One may speculate about the possible reasons why the RN rates after SRS for lesions > 20 mm were so high in the present study. One possible explanation is the low sample size of only eleven patients in this subgroup. Moreover, 7 of these patients died within 10 months following SRS and were, therefore, removed from the analyses of RN at 1 year and 2 years. Therefore, the very few cases of RN (*n* = 3) resulted in high RN rates. In addition to the study by Minniti et al., other studies found that SRS was associated with a higher risk of RN than FSRT [9,30]. In contrast, in the study of Hirata et al., who treated up to 10 brain metastases, the rates of symptomatic RN were not significantly different after SRS and FSRT (hazard ratio: 1.35, 95% confidence interval: 0.35–0.67, p = 0.67) [20]. Thus, this point also needs further investigation.

When using SRS with a higher BED for brain metastases ≤ 20 mm, the rates of symptomatic RN in our study were only 4% at 1 and 2 years. These rates were at the lower end of the range of 4–16% found in previous studies, which further supports the feasibility of SRS with 19–20 Gy for lesions ≤ 20 mm [12,14,15,17,18,19,20,21]. However, during the interpretation of our results, the limitations of this study must be considered, particularly the retrospective design including the risk of hidden selection biases, the small sample size in the subgroup of patients with brain metastases > 20 mm in maximum diameter (particularly in patients treated with SRS), and the difference regarding the distribution of patient characteristics between the SRS and FSRT groups. Therefore, this study must be considered to be a hypothesis-generating pilot study that may contribute to the design of future prospective randomized trials.

## 5. Conclusions

Given the limitations of this study, FSRT with a higher BED (>49 Gy_12_) appeared to be a safe option for brain metastases > 20 mm. SRS may not be optimal for metastases > 20 mm, but appears to be safe for lesions ≤ 20 mm. Prospective trials are warranted to define the optimal dose-fractionation regimen of stereotactic radiotherapy for brain metastases > 20 mm.

## Figures and Tables

**Table 1:** Distributions of patient characteristics in SRS and FSRT groups in the entire cohort.

Characteristic	Number of Patients and Proportion (%)		*p*-Value
	SRS (*n* = 100)	FSRT (*n* = 69)	
Biologically effective dose			**<0.001**
49–57 Gy_12_	100 (100)	39 (57)	
63–67 Gy_12_	0 (0)	30 (43)	
Year of treatment			0.92
2011–2016	37 (37)	25 (36)	
2017–2022	63 (63)	44 (64)	
Pre-RT systemic treatment			**0.004**
No	33 (33)	38 (55)	
Yes	67 (67)	31 (45)	
Age at RT			0.84
≤66 years	52 (52)	37 (54)	
≥67 years	48 (48)	32 (46)	
Gender			0.33
Female	54 (54)	32 (46)	
Male	46 (46)	37 (54)	
Karnofsky performance score			**<0.001**
≤80	27 (27)	41 (59)	
≥90	73 (73)	28 (41)	
Type of primary tumor			0.24
Lung cancer	38 (38)	33 (48)	
Melanoma	34 (34)	24 (35)	
Other types	28 (28)	12 (17)	
Number of brain metastases			**<0.001**
1	87 (87)	42 (61)	
2–4	13 (13)	27 (39)	
Main metastatic site(s)			0.18
Supratentorial–peripheral	60 (60)	50 (72)	
Supratentorial–central	15 (15)	5 (7)	
Infratentorial	25 (25)	14 (20)	
Maximum size of brain metastases			**0.002**
≤20 mm	89 (89)	48 (70)	
>20 mm	11 (11)	21 (30)	
Metastases outside the brain			0.46
No	22 (22)	12 (17)	
Yes	78 (78)	57 (83)	

SRS: single-fraction stereotactic radiosurgery; FSRT: fractionated stereotactic radiotherapy; RT: radiotherapy.

**Table 2:** Distributions of characteristics in SRS and FSRT groups (patients with ≥1 lesion >20 mm).

Characteristic	Number of Patients and Proportion (%)		*p*-Value
	SRS (*n* = 11)	FSRT (*n* = 21)	
Biologically effective dose			0.066
49–57 Gy_12_	11 (100)	14 (67)	
63–67 Gy_12_	0 (0)	7 (33)	
Year of treatment			0.37
2011–2016	8 (73)	10 (48)	
2017–2022	3 (27)	11 (52)	
Pre-RT systemic treatment			0.47
No	4 (36)	11 (52)	
Yes	7 (64)	10 (48)	
Age at RT			0.46
≤65 years	4 (36)	12 (57)	
≥66 years	7 (64)	9 (43)	
Gender			0.91
Female	5 (45)	10 (48)	
Male	6 (55)	11 (52)	
Karnofsky performance score			**0.002**
≤80	1 (9)	15 (71)	
≥90	10 (91)	6 (29)	
Type of primary tumor			0.095
Lung cancer	2 (18)	12 (57)	
Melanoma	5 (45)	4 (19)	
Other types	4 (36)	5 (24)	
Number of brain metastases			>0.99
1	7 (64)	12 (57)	
2–4	4 (36)	9 (43)	
Main metastatic site(s)			0.10
Supratentorial–peripheral	5 (45)	17 (81)	
Supratentorial–central	4 (36)	2 (10)	
Infratentorial	2 (18)	2 (10)	
Metastases outside the brain			0.31
No	3 (27)	2 (10)	
Yes	8 (73)	19 (90)	

SRS: single-fraction stereotactic radiosurgery; FSRT: fractionated stereotactic radiotherapy; RT: radiotherapy.

**Table 3:** Distributions of characteristics in SRS and FSRT groups (patients with all lesions ≤ 20 mm).

Characteristic	Number of Patients and Proportion (%)		*p*-Value
	SRS (*n* = 89)	FSRT (*n* = 48)	
Biologically effective dose			**<0.001**
49–57 Gy_12_	89 (100)	25 (52)	
63–67 Gy_12_	0 (0)	23 (48)	
Year of treatment			0.87
2011–2016	29 (33)	15 (31)	
2017–2022	60 (67)	33 (69)	
Pre-RT systemic treatment			**0.007**
No	29 (33)	27 (56)	
Yes	60 (67)	21 (44)	
Age at RT			0.75
≤66 years	47 (53)	24 (50)	
≥67 years	42 (47)	24 (50)	
Gender			0.30
Female	49 (55)	22 (46)	
Male	40 (45)	26 (54)	
Karnofsky performance score			**0.004**
≤80	26 (29)	26 (54)	
≥90	63 (71)	22 (46)	
Type of primary tumor			0.23
Lung cancer	36 (40)	21 (44)	
Melanoma	29 (33)	20 (42)	
Other types	24 (27)	7 (15)	
Number of brain metastases			**<0.001**
1	80 (90)	30 (63)	
2–4	9 (10)	18 (38)	
Main metastatic site(s)			0.50
Supratentorial–peripheral	55 (62)	33 (69)	
Supratentorial–central	23 (26)	12 (25)	
Infratentorial	11 (12)	3 (6)	
Metastases outside the brain			0.94
No	19 (21)	10 (21)	
Yes	70 (79)	38 (79)	

SRS: single-fraction stereotactic radiosurgery; FSRT: fractionated stereotactic radiotherapy; RT: radiotherapy.

## Data Availability

The data analyzed for this article cannot be shared according to data protection regulations. Only the evaluation of anonymized data is allowed according to the decision of the responsible ethics committee.
